# Treatment of SARS-CoV-2 pneumonia with favipiravir: early results from the Ege University cohort, Turkey

**DOI:** 10.3906/sag-2008-33

**Published:** 2021-06-28

**Authors:** Hüseyin Aytaç ERDEM, Pervin KORKMAZ EKREN, Derya ÇAĞLAYAN, Meltem IŞIKGÖZ TAŞBAKAN, Tansu YAMAZHAN, Mehmet Sezai TAŞBAKAN, Abdullah SAYINER, Deniz GÖKENGİN

**Affiliations:** 1 Department of Infectious Diseases and Clinical Microbiology, Faculty of Medicine, Ege University, İzmir Turkey; 2 Department of Chest Diseases, Faculty of Medicine, Ege University, İzmir Turkey

**Keywords:** SARS-CoV-2, favipiravir, COVID-19, coronavirus, pandemic, antiviral therapy

## Abstract

**Background/aim:**

The aim of this descriptive article is to share the experience in Ege University, Turkey with favipiravir in the treatment of severe SARS-CoV-2 pneumonia.

**Materials and methods:**

This retrospective descriptive study included patients diagnosed with COVID-19 who presented with or developed severe pneumonia.

**Results:**

Forty patients who completed a full course (at least 5 days) of favipiravir were included in the study. At baseline, 30 (75%) patients required treatment for respiratory distress. Thirty-three patients (82.5%) were discharged from the hospital with full recovery, 6 patients (15%) died and 1 case (2.5%) was still at the intensive care unit (ICU) when this paper was written.

**Conclusion:**

This study provides relevant information for the treatment of COVID-19, suggesting that favipiravir was associated with significant clinical and laboratory improvements in the majority of the patients, is a safe drug with no serious side effects and would merit further investigation.

## 1. Introduction

The large-scale pandemic due to the severe acute respiratory syndrome coronavirus (SARS-CoV-2), which started in November 2019 in Wuhan, China has caused 4,425,485 confirmed cases of coronavirus infectious disease (COVID-19) in 216 countries, and 302,059 confirmed deaths as of May 16th, 2020. Republic of Turkey Ministry of Health (2020). Guidelines for the Management of Adults with COVID-19 [online]. Website https://covid19bilgi.saglik.gov.tr/depo/rehberler/COVID-19_Rehberi.pdf [accessed 01 May 2020].

SARS-CoV-2 is reported to be transmitted from human to human by droplets and to cause lower respiratory tract infection, which may result in severe respiratory distress and death [1]. Treatment of COVID-19 is challenging as there is no proven treatment strategy yet. Small-scale, randomized controlled trials and observational cohort studies are available for several drugs with controversial results. Chloroquine/hydroxychloroquine, azithromycine, lopinavir/ritonavir, favipiravir, and remdesivir, are some of which have been shown to have in vitro activity against MERS-CoV, SARS-CoV, other coronaviruses and various other viruses. These drugs are among those that are most promising, but with controversial results in clinical studies that are difficult to interpret [2–17].

While it is difficult to design a treatment approach without proven benefit of a drug or a treatment regimen, the severity of the situation calls for urgent action. Considering the emergency state we are all in, every piece of data counts and contributes to the knowledge and understanding of the pandemic, helping us shape our approach to treating the patients. The aim of this descriptive article is to share the experience in Ege University, Turkey with favipiravir in the treatment of severe SARS CoV-2 pneumonia by providing real-world data while waiting for the solid evidence to build up.

## 2. Materials and methods

This retrospective descriptive study included patients diagnosed with COVID-19 who were hospitalized in the Department of Clinical Microbiology and Infectious Diseases and the Department of Chest Diseases, at Ege University Faculty of Medicine from March 23rd to April 26th 2020. The diagnosis was made by a positive nucleic acid amplification test (NAAT) from nasopharyngeal swabs (Coyote Biosciences, San Jose, CA, USA) and radiologic findings in line with COVID-19 pneumonia [19]. Patients who presented with or developed severe pneumonia [tachypnea (> 30 breaths/min) and/or hypoxia (SpO2 < 90% on room air) and/or bilateral diffuse ground glass infiltrations] with no response to first-line treatment with HCQ (± azithromycin) and started favipiravir were included. 

The treatment approach was in line with the recommendations of the Guidelines for the Management of Adults with COVID-19 produced by the Turkish Ministry of Health.1

Favipiravir 200 mg tablets were administered orally starting with a loading dose of 1600 mg bid, followed by 600 mg bid daily for 5 to 7 days. 

For each patient, demographic characteristics, underlying conditions, clinical signs and symptoms, radiologic and laboratory findings, oxygen requirement, and adjunctive treatments used were recorded. Response to favipiravir treatment was assessed by comparison of baseline and day 4 of treatment in terms of the level of respiratory distress and laboratory parameters [white blood cell count and differential, lactate dehydrogenase (LDH), C-reactive protein (CRP), ferritin, fibrinogen, d-dimer, and procalcitonin]. 

Oxygen therapy was classified as oxygen delivery via face mask, continuous positive airway pressure (CPAP) and invasive mechanical ventilation (IMV). Respiratory distress level and chest X-ray/high resolution computed tomography (HRCT) findings were evaluated at baseline prior to initiation of favipiravir and on day 4 of the treatment. Patients who did not require oxygen therapy before or after favipiravir treatment were defined as the “ambient air” group. Patients whose oxygen requirement did not change from baseline until day 4 were classified as the “no change” group. Patients whose oxygen requirement reduced from baseline to day 4 were grouped as “improvement”, and those whose oxygen requirement increased were defined as “impairment”. Treatment was considered successful for patients who were discharged from the hospital with no symptoms, improvement in chest radiology, and no respiratory distress. In addition, adverse events and serious adverse events that developed during favipiravir treatment were recorded. 

### 2.1. Statistical analysis

Data was analyzed using SPSS v.18.0 (SPSS Inc., Chicago, IL, USA). Numbers and percentages were used for categorical variables. Mean values with standard deviations, and median values with minimum and maximum values were calculated for continuous variables. Continuous variables were tested for agreement with normal distribution. Since all variables did not show normal distribution in graphical analyses and normalization tests, and considering the size of the study population, nonparametric methods were preferred for comparisons. The Wilcoxon signed ranks test was used to analyze the change in repeating measurements. A type-1 error α was determined as 0.05 and was tested. A value of p < 0.05 was considered statistically significant and was represented with a box pilot graphic.

## 3. Results

Out of a total of 559 patients admitted to the hospital, 40 patients who completed a full course (at least 5 days) of the drug during the study period were included in the study. More than half (58%) of the patients were male. The mean age was 55.58 ± 13.49 (26–79 years). All patients were confirmed COVID-19 cases with positive nasopharyngeal swabs. Two thirds of the patients had at least one underlying disease, and the most common underlying conditions were cardiovascular disease and diabetes mellitus. The most common clinical symptoms were fever and cough; almost all (95%) patients had more than one symptom. Baseline demographic characteristics, comorbidities, clinical signs and symptoms, as well as respiratory distress levels are shown in Table 1.

**Table 1:** Demographic and baseline (prior to initiation of favipiravir) characteristics of patients.

Characteristics	n = 40 (%)
Age groups	
≤50 year	12 (30)
50 to < 70 year	22 (55)
≥70 year	6 (15)
Sex	
Male	23 (58)
Female	17 (42)
Health care workers	6 (15)
Underlying medical conditions	25 (63) (at least one)
Hypertension	12 (30)
Diabetes mellitus	11 (28)
Coronary artery disease	5 (13)
Malignancy	3 (8)
Hypothyroidism	3 (8)
Asthma	2 (5)
Contact with confirmedCOVID-19 infection	17 (43)
Signs and symptoms	
Fever	34 (85)
Cough	29 (73)
Dyspnoea	15 (38)
Sore throat	11 (28)
Fatigue	10 (25)
Sputum production	7 (18)
Diarrhea	7 (18)
Oxygen support status at baseline	
Face mask	19 (47.5)
Ambient air	10 (25)
CPAP	7 (17.5)
Mechanical ventilation	4 (10)
Need for inotropes or vasopressors during favipiravir treatment	4 (10)

Twenty (50%) patients were managed in the intensive care unit (ICU); 5 patients were admitted directly to the ICU at presentation and 15 patients were transferred to the ICU from the clinic ward due to clinical worsening. The mean duration of stay at the ICU was 9.31 (1–19) days. At baseline, 30 (75%) patients required treatment for respiratory distress, Table1. While 9 patients (22.5%) maintained respiration at ambient air before and after favipiravir treatment, oxygen requirement increased in 13 (32.5%) patients, decreased in 12 (30%) and did not change in 6 (face mask/nasal cannula, CPAP or IMV) (15%) on day 4 of favipiravir treatment. The change in oxygen requirement after favipiravir treatment did not differ according to age or sex (p > 0.05). On day 4 of favipiravir treatment, radiologic findings improved or did not deteriorate in 42.5% of the cases, and deteriorated in 42.5%; no radiologic follow-up was available in 15%. 

**Figure 1:**
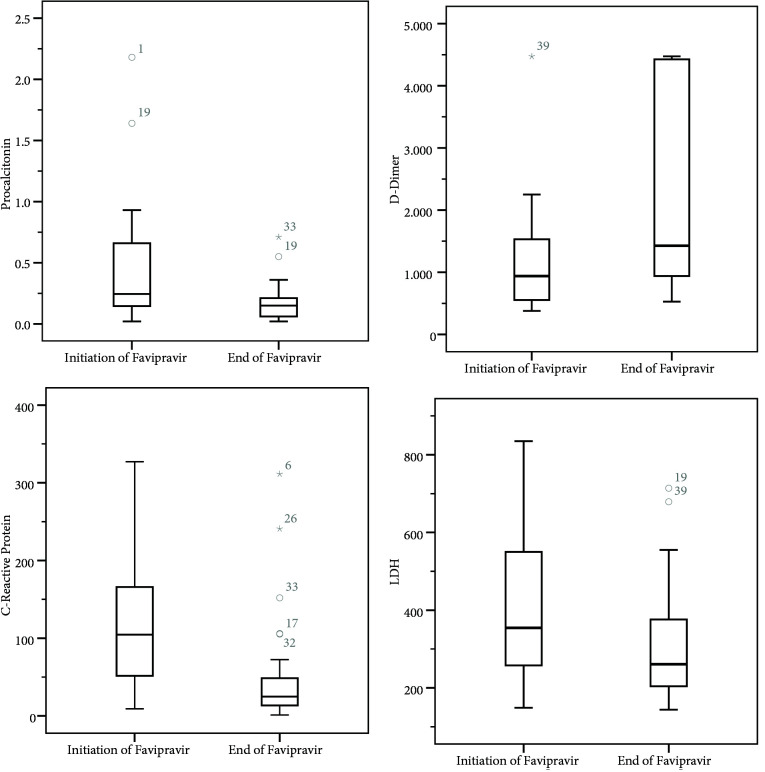
Distribution of statistically significant laboratory results.

Table 2 includes laboratory findings before and 24 h after the termination of favipiravir treatment. C-reactive protein, procalcitonin, LDH and d-dimer levels were elevated before favipiravir treatment; while CRP, procalcitonin and LDH levels decreased significantly, d-dimer levels continued to increase after favipiravir treatment. Figure 1 shows the distribution of statistically significant laboratory results.

**Table 2:** Laboratory findings of patients with COVID-19.

Laboratory results	Initiation of favipravir median (min-max)	End of favipravirmedian (min-max)	p value
Leukocyte (cell/mm3) (n = 37)	6070 (3320–20770)	6690 (3830–17130)	0.23
Neutrophil (cell/mm3) (n = 37)	4710 (1880–19570)	4600 (1790–15960)	0.47
Lymphocytes (cell/mm3) (n = 37)	890 (290–2410)	1310 (390–3470)	0.001
C-reactive protein (mg/L) (n = 37)	104 (9–327)	24 (1–311)	<0.001
Procalcitonin (mg/L) (n = 24)	0.24 (0.02–2.18)	0.15 (0.02–0.71)	0.003
Ferritin (µg/L) (n = 24)	696 (86–3498)	565 (103–4188)	0.97
D-dimer (mg/L) (n = 32)	938 (377–4472)	1426 (527–4473)	0.001
Lactate dehydrogenase (U/L) (n = 26)	355 (149–835)	261 (144–714)	0.001
Alanine aminotransferase (U/L) (n = 38)	29 (5–258)	46 (6–198)	0.001
Aspartate aminotransferase ( U/L) (n = 38)	41 (6–269)	32 (9–149)	0.41

Adverse events developed in 5 patients (13%) during favipiravir treatment. All five had mild to moderate elevations in hepatic enzymes. Three of these cases also reported nausea and one case developed neutropenia. Adverse events were resolved spontaneously, no patient developed a serious adverse event and no patient discontinued favipiravir due to adverse events. While 17 patients required no adjunctive treatment, 23 patients received various combinations of favipiravir with tocilizumab, corticosteroid, intravenous immunoglobulin (IVIG), and plasma transfer. Low molecular weight heparin (LMWH) was administered to 34 patients (85%). 

**Figure 2:**
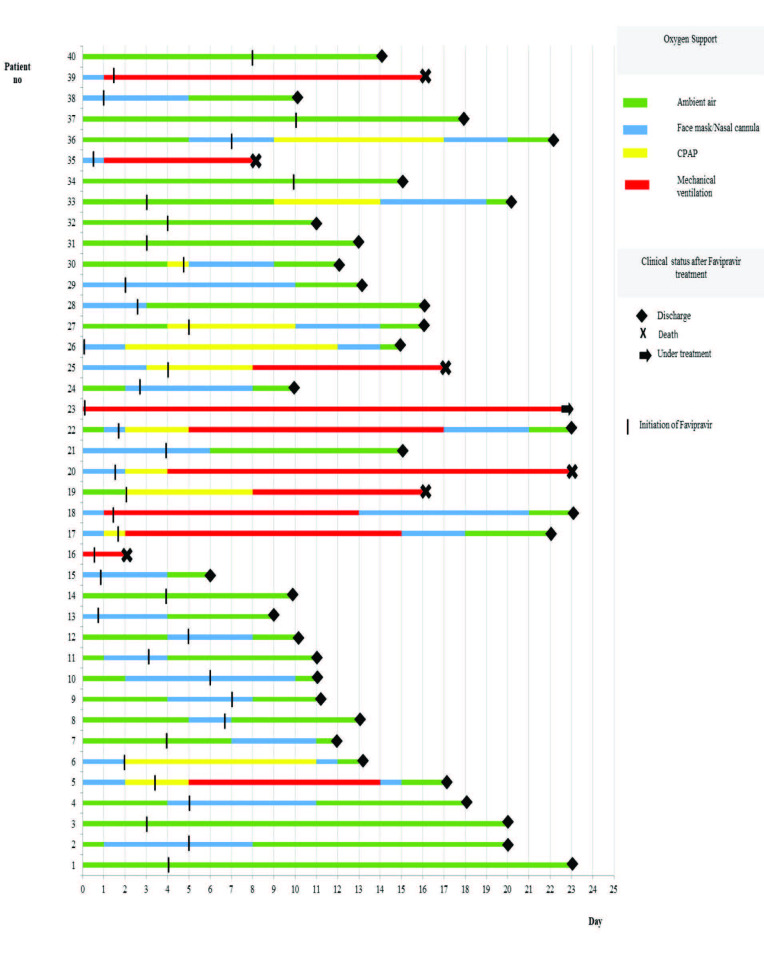
Oxygen support status before and after favipiravir treatment, and outcomes of the patients.

Thirty-three patients (82.5%) were discharged from the hospital with full recovery, 6 patients (15%) died and 1 case (2.5%) was still at the ICU when this paper was written. Of those who died, 4 were older than 65 years and the remaining two were 52 and 64 years old. All patients who died had started favipiravir when they were already in the ICU, had at least one serious comorbidity excluding one 64-year old case, and had received various combinations of tocilizumab (n = 5), corticosteroid (n = 5), and plasma transfer (n = 1). Figure 2 summarizes the method of treatment for respiratory distress before and after favipiravir treatment, and the outcome of each patient. 

Overall 30/40 patients were screened with nasopharyngeal swabs after the completion of favipiravir treatment before or after discharge at different time points. Twenty-seven patients were negative. Of the remaining 3 patients with positive results, 1 recovered and was discharged from the hospital without a negative result, 2 died before a negative result.

## 4. Discussion

This study is the first from Turkey and one of the few globally on favipiravir use in the treatment of COVID-19 patients with severe pneumonia. Despite its retrospective nature, it may aid in providing insight into whether favipiravir deserves further investigation and may be an option for treating COVID-19 patients by presenting real world data. 

COVID-19, an acute respiratory condition due to SARS-CoV-2 has spread rapidly resulting in a pandemic with devastating effects within a few months since November 2019. Unlike its predecessor SARS, asymptomatic cases are present, the viral load peaks earlier and commonly before the patient is symptomatic, transmission rate is higher, and attack rates are variable in different geographic regions in SARS-CoV-2 infection, which makes containment difficult and creates an emergency for rapid action [18–23]. Although more than 80% of the patients with COVID-19 have mild pneumonia, which usually resolves rapidly, 13%–18% of the cases develop severe pneumonia and 4%–9% are reported to be critically ill [23,24]. Individuals with diabetes and hypertension were reported to have a higher likelihood of developing severe infection (44.5% and 41.7%, respectively) and a higher case fatality rate [23,24]. The analysis of our cohort including severe COVID-19 cases revealed a relatively high age distribution and a high rate of comorbidities with hypertension and diabetes being the most common, in line with the results of other cohorts [25,26]. The outcome of severe COVID-19 is poor, mostly requiring hospitalization at the ICU and mechanical ventilation [26]. 

A major challenge in the COVID-19 pandemic is the lack of evidence on reliable treatment options. Although various agents are under investigation in ongoing clinical trials, the urgency of the situation has led scientists to use empirical treatments or drugs that have been investigated for other viruses in the past. Chloroquine and its derivative HQ, which are antimalarial drugs were among the most studied and recommended molecules for the treatment of COVID-19 based on earlier reports on their in vitro antiviral effects on SARS virus [27] and their antiinflammatory and immunomodulator effects [5]. Several clinical studies were undertaken to study the clinical success of CQ/HQ as monotherapy or coadministered with azithromycine with conflicting results [13,14,17,28]. A metaanalysis showed that although HCQ treatment compared to standard treatment had some clinical benefits, there was no difference in virologic cure, clinical progression or death rates [28].

Similarly, clinical studies reported controversial results with LPV/r [16,29,30]. A systematic review reported that no specific conclusion could be drawn for the efficacy of LPV/r in COVID-19 [31]. 

Favipiravir was shown to inhibit a wide array of RNA viruses such as the influenza virus, arenavirus, bunyavirus, flavivirus, and filoviruses [32] and to improve survival in patients with Ebola virus infection [33,34]. It was considered as a potential candidate for the treatment of COVID-19 and the urgency of the situation required its premature use without in vitro and animal studies [35]. Favipiravir was studied in a few randomized controlled studies with promising results. It was reported to significantly shorten the time to viral clearance and alleviate the symptoms of pneumonia, thereby improving the chest imaging compared to LPV/r [36]. It also had a significantly higher recovery rate compared to umifenovir, and shortened the time with fever and respiratory symptoms even in patients with hypertension and diabetes, albeit with no statistically significant difference between the two drugs in terms of requirement for oxygen support and noninvasive mechanical ventilation [37]. 

Favipiravir did not become a favored drug globally and was recommended and used only in Japan, China and Turkey during the pandemic [38].1 Since the beginning of the epidemic the Guidelines for the Management of Adults with COVID-19 produced by the Turkish Ministry of Health has suggested the addition of favipiravir or LPV/r in COVID-19 patients who do not respond to first-line treatment with HCQ and develop severe pneumonia, and direct initiation of favipiravir or LPV/r ± HCQ to those that present with severe pneumonia.1 Th treatment approach in the Ege University was to initiate favipiravir in severe cases after, during or in addition to HQ treatment. The main reason to prefer favipiravir over LPV/r was previous experience with LPV/r in people living with HIV in terms of severe adverse events sometimes leading to permanent discontinuation of the drug. Despite its retrospective design the results of our study suggest a high rate of improvement in severe COVID-19 patients. More than 80% of the patients, including several who received noninvasive and invasive mechanical ventilation were discharged from the hospital with full recovery. The most notable finding was an early response in approximately a third of the patients in terms of reduced oxygen requirement with further improvement within time. In addition, all cases but one that required no oxygen support at baseline maintained their status after favipiravir treatment. The favorable outcome in the majority of the cases ()resulting with full recovery was promising, although several patients recovered at later stages. The poorest response was among those who were already on IMV or CPAP when favipiravir was initiated suggesting that early initiation of the drug before or right after respiratory distress or radiologic deterioration develops might be most beneficial. Favipiravir treatment did not seem to have a major effect on the resolution of radiologic findings at early stages in contrast to the findings of Cai et al. This may be attributed to the severity of lung involvement in our cases mostly requiring oxygen support whereas those with severe pneumonia were excluded in the study by Cai et al. [36]. 

Favipiravir proved to have a favorable safety profile both for COVID-19 and influenza treatment at various dosing regimens and compared to umifenovir and LPV/r, the most common adverse events being elevations in liver enzymes and bilirubin as well as gastrointestinal disturbances, and elevations in uric acid [36,37,39]. Our cohort confirms the safety of the drug even in severe pneumonia cases with multiple comorbidities using other adjunctive therapies. There was no serious adverse event that resulted with discontinuation of the drug.

The severity of the COVID-19 pneumonia is associated with a cytokine storm with increased levels of proinflammatory cytokines and ferritin causing severe inflammation and hypoxia [40–42]. In addition, the development of thromboembolic events worsens the situation, sometimes even leading to death. Elevated d-dimer level was suggested to be an independent risk factor for death [43]. Thus, adjunctive treatment approaches in addition to antiviral treatment to alleviate the inflammation such as anticytokinic biological agents, corticosteroids and IVIG in addition to anticoagulants, and especially LMWH were recommended [40,42,44]. The antiinflammatory effects of CQ/HCQ and azithromycine have also been suggested to contribute to the management of inflammation [5]. The analysis of our patients reveal increased levels of inflammation markers, ferritin, and d-dimer, which are in line with the severity of the condition. Various adjunctive treatments were used to address the inflammation and coagulopathy in our patients. While inflammation markers reduced significantly after the relevant treatment approach, high d-dimer levels maintained despite the use of heparin and this may be related to the course of the disease. Tang et al. reported that elevated d-dimer levels were significantly associated with higher mortality and although nonsignificant, 28-day mortality was lower in heparin users compared to nonusers. Timely and correct use of corticosteroids in conjunction with ventilator support may be life-saving in severe COVID-19 patients by preventing ARDS [41,45,46]. It was not possible to analyze the association of inflammation and coagulation markers to patient outcome due to the small size of the study group. 

This study has several limitations:

(1) This is not a randomized controlled study and its level of evidence is low.

(2) The study group was too small to make detailed statistical analyses.

(3) It was difficult to define the role of favipiravir in the recovery of the patients because many additional adjunctive treatments were administered.

(4) Patients were not followed-up for the duration of time required for viral clearance.

However, despite these limitations, this study provides relevant information for the treatment of COVID-19, suggesting that favipiravir was associated with significant clinical and laboratory improvements in the majority of the patients and is a safe drug with no serious side effects. Currently there are several completed and ongoing clinical trials studying favipiravir [47] and in a time of emergency when drugs found ineffective in randomized clinical trials [48] are authorized for emergency use by drug agencies it would merit further investigation.2,3

## Informed consent

The study was approved by the Ege University Ethical Board (date: April 30th, 2020 number: 20-4.2T/42). Informed consent was submitted by all subjects when they were enrolled.
